# ^1^H-NMR Metabolic Profiling and Antioxidant Activity of Saffron (*Crocus sativus*) Cultivated in Lebanon

**DOI:** 10.3390/molecules26164906

**Published:** 2021-08-13

**Authors:** Hala Samaha, Nathalie Chahine, Anatoly Petrovich Sobolev, Luigi Menghini, Hassane Makhlouf

**Affiliations:** 1 Laboratory of Geosciences, Georesources and Environment, Faculty of Sciences II, Lebanese University, Fanar 1202, Lebanon; halsam@ul.edu.lb (H.S.); drhassanemakhlouf@yahoo.fr (H.M.); 2 Faculty of Public Health, Lebanese University, Fanar 1202, Lebanon; nathaliechahine@hotmail.com; 3 Institute for Biological Systems, Magnetic Resonance Laboratory “Segre-Capitani”, National Research Council (CNR), Via Salaria Km 29.300, 00015 Monterotondo, Italy; 4 Department of Pharmacy, Botanic Garden “Giardino dei Semplici”, Università degli Studi “Gabriele d’Annunzio”, Via dei Vestini 31, 66100 Chieti, Italy

**Keywords:** saffron, NMR, Lebanon, metabolic profiling, geographical classification, microwave-assisted extraction

## Abstract

Despite the beneficial health properties shown by Lebanese saffron, its qualitative and quantitative composition has never been investigated before. In the present study, NMR spectroscopy, together with antioxidant activity assays, were applied to evaluate the chemical composition of saffron samples of different geographical origins (Lebanon, Italy, Iran, and India) and to categorize the Lebanese saffron for the first time. The distinction between Lebanese saffron and that produced in other countries was attributed to its higher linolenic and linoleic fatty acids, glucose and picrocrocin contents. Moreover, spices produced in three different regions of the Lebanese territory have been clearly differentiated. Saffron cultivated in the Qaa region displayed a high glucose, fatty acids and polyphenols content, whereas Hermel saffron exhibited the largest rate of picrocrocin and glycosylated carotenoids. Finally, samples from Baalbeck showed lower rates for the majority of metabolites. Moreover, Lebanese saffron showed a high antioxidant activity in ABTS and DPPH assays. A low dose of saffron extract (10 µg/mL) inhibited the growth of human lung adenocarcinoma cells, probably due to the high polyphenolic content. This study highlights the quality and peculiarity of Lebanese saffron cultivated in Northern Beqaa district and allows for a good discrimination between spices produced in relatively close territory.

## 1. Introduction

Saffron has been widely used as a spice for flavoring and coloring food since ancient times. It was also recognized for its medicinal and pharmacological activities, including anti-tumor [1], antioxidant [2] and anti-inflammatory properties, and even in the treatment of anxiety and schizophrenia [3]. This valuable spice derives from the flowers of *Crocus sativus* L. (Iridaceae); more precisely, from the stigmas, which are hand-picked during autumn and then dried, giving the spice a red-orange color. The laborious harvesting process and its limited production explain the high price of this spice. For this reason, the adulteration of saffron is frequently encountered, either by the addition of other saffron flower parts (tepals and styles) or by mixing with other plant products [4,5]. In this regard, is interesting to note that only stigmas, which represent less than 5% of the flower weight, are used, while other high-quality parts, such as tepals and stamens, are commonly considered by-products, and were only recently reconsidered as sources of healthy bioactive extracts [6,7].

The chemical composition of saffron has been thoroughly investigated. Many metabolites have been separated and analyzed using Gas Chromatography (GC) and GC coupled to Mass Spectrometry (GC/MS), whereas non-volatiles were analyzed using HPLC. More than 150 volatiles and aromatic compounds have been identified [8]. However, three major metabolites are responsible for saffron quality: (i) picrocrocin, a monoterpene glycoside responsible for the bitter flavor of saffron, (ii) safranal, monoterpenoid that contributes to the characteristic odor and (iii) crocins, a group of glycosides of C_20_ carotenoid aglycone crocetin, giving the golden color to this spice [8].

In order to obtain a major number of metabolites from the stigmas, conventional extractions with solvents were generally used. Recently, microwave-assisted saffron extraction in methanol has been applied successfully to *C. sativus* [9]. This technique permitted an overall better isolation of secondary metabolites, due to the entire swelling of the subcellular structures under microwave irradiation, leading to deeper penetration of the solvent [10]. This extraction method has many advantages, as it requires a minimal amount of the expensive dried stigmas, and reduces solvent volume and extraction time. Furthermore, pressurized and sealed vials reduce the loss of volatile components and allow for the strict control of multiple parameters (temperature, pressure and irradiation power). Nevertheless, in the case of saffron, both types of extraction (microwave-assisted and conventional extraction in methanol) gave similar results in terms of metabolite profile composition, as evidenced by NMR analysis [9].

The chemical composition and the quality of saffron depend on many factors such as harvesting and processing, different drying procedures, edaphic and climatic conditions. Thus, it is substantially based on the geographical origin of the plant. Different techniques were used to study the relationship between saffron composition and its geographical origin, such as chromatographic techniques [11], near-infrared [12,13] and mid-infrared spectroscopy [14] or multi-element (H, C, N) stable isotope analysis [15]. Recently, the nuclear magnetic resonance (NMR) technique has emerged as a tool to classify saffron samples on the basis of their geographical origin and to detect saffron adulteration [16]. The NMR technique has proven useful for origin discrimination purposes among Italian, Greek, Turkish, Spanish [9], and Iranian saffron samples [16].

Saffron is attractive as a functional food due to its potential beneficial effects on different human cancers and its chemopreventive properties [17]. Saffron is currently recognized for its antitumor properties, and its cytotoxic effect has been studied in different cancer cell lines [18] including the A549 lung cancer cell line [19]. The A549 cell line consists of hypotriploid alveolar basal epithelial cells and is the most used human lung cancer cell line for both basic research and drug discovery [20]. It is used for a wide range of research applications including the biological activity tests of different extracts and evaluation of their potential to induce the cytotoxic effect. Consequently, the A549 cell line was chosen in this study to assess the biological activity of saffron extracts from samples of different geographical origins.

Recently, Lebanese saffron has drawn increasing attention due to its high number of polyphenols and antioxidant activity [2,21]. These studies indicated that Lebanese saffron possesses a powerful scavenger potential against free radicals generated in vitro, and attenuates cardiac cytotoxicity induced by an anthracycline antibiotic. However, the qualitative and quantitative composition of Lebanese saffron has never been investigated to date. Saffron was introduced to Lebanon in 1999 and cultivated in the Beqaa region to substitute illicit crops. At present, this culture is gaining in importance throughout the country. To evaluate the quality and the variability of saffron composition among Lebanese regions, we characterized secondary metabolites of different Lebanese samples using NMR. Additionally, a comparison of the ^1^H metabolic profiling of Lebanese saffron samples with those of other geographical origins was carried out. In order to evaluate the quality of saffron samples from different production areas, antioxidant activity and total polyphenols content were also investigated. In addition, the biological activity of saffron samples, namely, their potential to induce a cytotoxic effect in human cancer cell lines, was assessed in vitro.

## 2. Results

### 2.1. Total Polyphenol Content and Antioxidant Activities

To carry out the total dosage of polyphenols, gallic acid was taken as a standard. The evaluation of total polyphenolic content, shown in Table 1, indicates that sample Leb1, taken from the region of Qaa, has the greatest value, which is approximately equal to the Iranian sample. The other regions showed a slightly decreased polyphenolic content, especially sample Leb2 (Hermel region) and Leb3 (Baalbeck region), but this variation was not statistically significant.

To best assess antioxidant activity, the general consensus is to use more than one technique, since a reliable and convenient technique to evaluate antioxidants in biological samples is lacking [22]. We have chosen two widely used and inexpensive methods to evaluate the antioxidant content and to compare the accuracy of the results of both assays. First, the antioxidant capacity of samples was investigated by the ABTS radical scavenging activity test, using Trolox as a standard. The results are represented as a percentage of the control value. In this assay, the most potent radical scavengers were Leb1 and the Iranian sample, followed by Leb2 and the Indian sample and, finally, Leb3. The samples differ significantly in terms of their ABTS radical cation scavenging capacities, and this difference seems to be related to the sample region. As seen in Table 1, Lebanese saffron extract (Leb1) and the Iranian sample showed a higher antioxidant capacity than other samples in terms of DPPH. Furthermore, the antioxidant activity is significantly different between samples from different geographical origins. Both tests confirm that sample Leb1 has the highest free radical scavenging capacity among the Lebanese samples.

### 2.2. Effect of Saffron Origin on Cell Viability

The cell survival percentage of A549, human lung adenocarcinoma cells, receiving saffron treatment was measured using an MTT assay, based on the conversion of MTT to formazan crystals by mitochondrial dehydrogenases. As shown in Table 2, cells were incubated with different concentrations of saffron. The results showed significantly reduced cell survival in A549 cells exposed to saffron in a dose-dependent manner. Thus, 10 μg/mL of the saffron extract led to 75% cell viability (versus control), whereas 40 μg/mL decreases the survival of the cancer cell line down to 25%. We notice that Lebanese and Iranian saffron are almost equivalent in their capacity to induce cell inhibition in adenocarcinoma cells.

### 2.3. Saffron Metabolite Profile by High Resolution NMR

Saffron samples of different origins, see Table 3, were subjected to an MW-extraction in deuterated methanol before an NMR analysis according to the previously developed and optimized method [9]. Metabolite profiling obtained by NMR consisted of the quantification of a number of compounds, which were most abundant in the extract and previously identified in NMR spectra [9], belonging to different classes, such as fatty acids, sugars, glycosylated carotenoids, and terpenoids.

**Figure 1:**
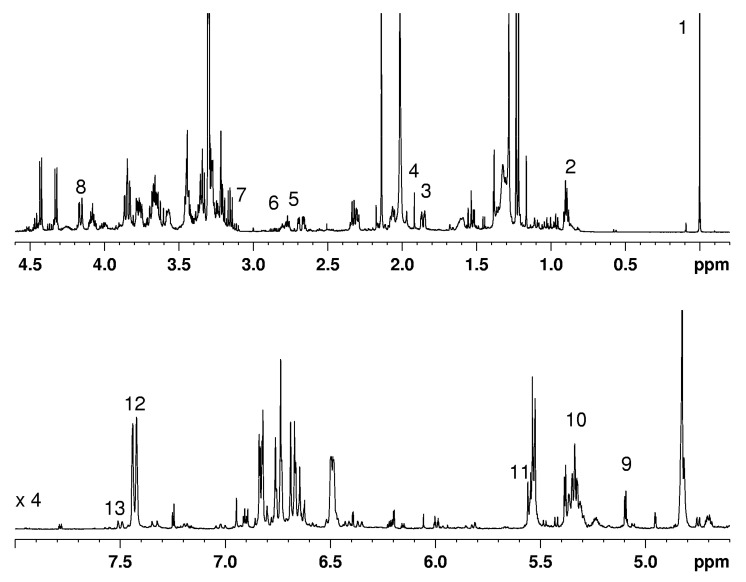
^1^H NMR spectrum of Lebanese saffron extract in CD_3_OD at 27 °C. Assignment: (1) CH_3_ of TSP; (2) CH_3_ of FA; (3) CH_2_-5 PCROC; (4) CH_3_ AcOH; (5) CH_2_-11 C18:2; (6) CH_2_-11,14 C18:3; (7) CH-2 βGLC; (8) CH_2_-6 GB-Croc; (9) CH-1 αGLC; (10) CH=CH UFA; (11) CH-1 βLC-Croc; (12) CH-10,10′ all trans-crocin DBtCroc; (13) CH-10 cis-crocin DBcCroc.

**Figure 2:**
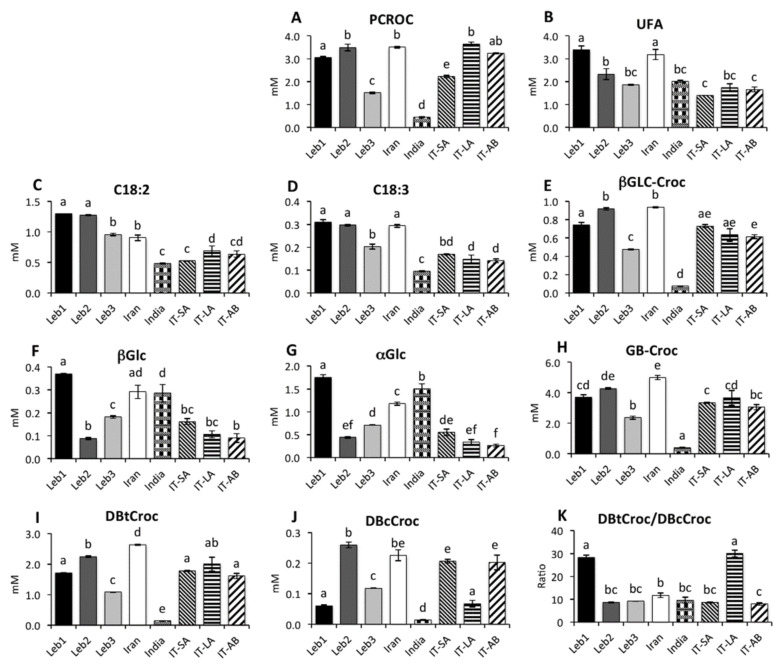
Bar charts relative to concentration (in mM, mean ± SD) of selected components in saffron CD_3_OD extracts obtained by MAE method. (**A**) Picrocrocin, (**B**) unsaturated fatty acid, (**C**) Linoleic fatty acid, (**D**) Linolenic fatty acid, (**E**) β-D-glucosylcrocins, (**F**) α-Glucose, (**G**) β-Glucose, (**H**) β-D-gentiobiosylcrocins, (**I**) All-trans-crocetin, (**J**) 13-cis-crocetin, (**K**) All-trans-crocetin/13-cis-crocetin ratio. Different single letters on the top of the bars (a, b, c, d, e, f) indicate significantly different mean values at *p* < 0.05.

Figure 1 shows a typical NMR spectrum of Lebanese saffron extract and the signals selected for the quantification of saffron components. The characteristic components of genuine saffron spice, such as picrocrocin, and crocetin glycosides, are clearly observed in the spectra in Figure 1, indicating the high quality of the investigated samples. Figure 2 reports on the concentration of selected metabolites in saffron extracts of different origins. Picrocrocin (PCROC) content in Leb1 and Leb2 samples was comparable with those in Iranian and two Italian ones (IT-LA and IT-AB) and two times greater than those in Leb3. The highest concentration of picrocrocin was observed in IT-LA, Iran, and Leb2 samples, whereas the lowest one (eight times lower) was found in the Indian sample.

Other characteristic components of saffron, crocins, belong to a class of glycosylated carotenoids, which are mono- or diesters of the C_20_-dicarboxylic acid crocetin (8,8′-diapocarotene-8,8′-dicarboxylic acid) with two possible configurations of double bounds. In the most abundant crocetin (DBtCroc), all double bonds are in trans-configuration, whereas the less abundant isomeric form, 13Z-crocetin (DBcCroc), is also present. The two most abundant glycosides in crocins are β-D-gentiobiosyl and β-D-glucosyl. Considering that crocins were composed of different combinations of two glycosides and crocetins, four different NMR signals were selected to characterize the average content of different components: two signals from glycoside moieties and two from two crocetin isomers. Regarding the two crocetin isomers, about 90% of crocetin was the isomer with all double bonds in trans-configuration, but, as can be seen from Figure 2K, in Leb1 and IT-LA samples, the fraction of DBtCroc was even higher, reaching more than 95%. The high ratio of DBtCroc/DBcCroc was a characteristic marker of both Leb1 and IT-LA samples. The highest absolute content of DBtCroc was observed in the Iranian sample, followed by the Leb2 and IT-LA ones, whereas the Indian sample showed a very low concentration of DBtCroc, which was almost ten times lower. DBcCroc isomer content in Leb2 showed the highest concentration compared to other samples. Glycoside moieties of crocin followed a similar trend: the highest content of GB-Croc and βGLC-Croc was observed in Iranian samples followed by Leb2. Among the Lebanese samples, the most important proportion of crocins was obtained in Leb2 (Hermel), followed by Leb1 (Qaa), and lower amounts were found in Leb3 (Baalbeck).

The content of α- and β-anomeric forms of D-glucose was correlated due to the mutual interconversion of both tautomers, with prevailing α-isomer in equilibrium. The content of glucose was the highest in the Leb1 sample compared to other samples. Saturated and unsaturated fatty acids were also extracted in methanol. The contents of linoleic (C18:2), linolenic (C18:3) and total unsaturated fatty acids (UFA) were roughly correlated among the different samples. Leb1 and Leb2 were characterized by the highest content of both C18:2 and C18:3 among all the samples. Leb3 showed a lower content of these fatty acids.

### 2.4. PCA Analysis of Saffron Metabolite Profile

**Figure 3:**
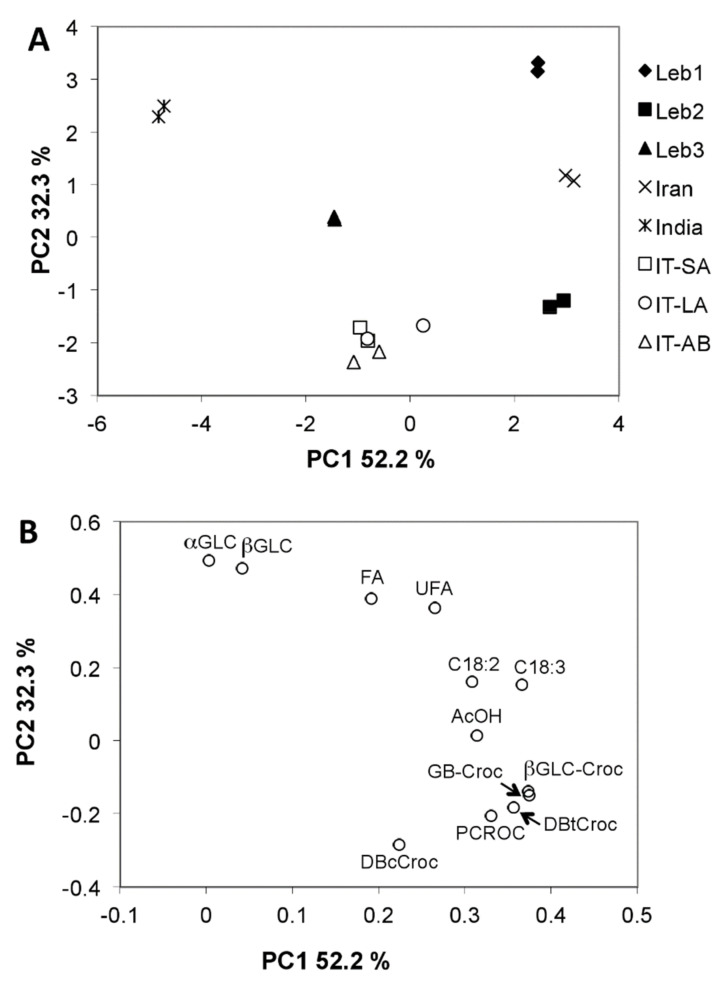
PCA scores (**A**) and loadings (**B**) plots of NMR metabolite profiling data.

To compare the metabolite profiling of saffron samples of different origins, a multivariate statistical approach was utilized. Principal component analysis (PCA) of NMR data was performed, as reported in Figure 3. PCA enables one to visualize the similarities and differences in multivariate space, projected in a two-dimensional graph to capture the principal features of the data. Figure 3A shows the scores plot, which includes two orthogonal dimensions showing 84.5% of total data variability. The loadings plot, Figure 3B, indicates the contribution of different variables in sample separation. It appears that the content of characteristic saffron components, picrocrocin and crocins, had the highest contribution in terms of PC1 scores. The Indian sample with the lowest picrocrocin and crocin content has the lowest PC1 score; Leb3 and some Italian samples are in the middle of the PC1 axis, whereas Leb1, Leb2 and Iranian samples show the highest PC1 scores. This order roughly corresponds to the content of crocins and, partially, picrocrocin in saffron samples. The low content of these components in the Indian sample indicates its low quality or the possibility of adulteration through the dilution of saffron with other colored substances, which probably originated from other plant species.

In the PCA scores plot, all Italian samples are grouped near to each other, indicating the similarity of their metabolite profiles, whereas the three Lebanese samples are quite distant and, therefore, each Lebanese location gives rise to a characteristic metabolite profile of correspondingsaffron sample that is distinct from the others. According to PCA, among the three Lebanese samples, Leb3 is characterized by lower picrocrocin and crocins contents, whereas Leb1 and Leb2 have higher levels of these components. The difference between Leb1 and Leb2 was observed due to the higher content of glucose and lower content of DBcCroc isomer in Leb1 compared to the Leb2 sample. The Iranian sample, located in the PCA in between Leb1 and Leb2 ones, shares similar metabolite profile characteristics with these two Lebanese samples.

## 3. Discussion

Two different extraction techniques were used, applying the same extraction solvent and almost the same solid-to-liquid ratio (50 vs. 12.5 g/L). The classical extraction aimed to maximize the phenolic compound extraction as bioactive compounds, using mild conditions to preserve them, whereas the MW-assisted extraction was previously optimized [9] to be suitable for an accurate, rapid and direct NMR analysis of the main metabolites and was proposed for commercial analyses of this expensive natural spice. According to the NMR study [9], both types of extraction provide similar results in terms of metabolite profile. Accounting for the fact that cell viability assay has to be performed in aqueous media, the direct, MW-assisted saffron extraction in methanol was not suitable. As a consequence, conventional extraction in methanol was used, followed by solvent evaporation and solubilization of extract in aqueous solutions.

The description of chemical constituents of Lebanese saffron, collected from three different regions, was reported for the first time in this study. Data collected from NMR analysis captured essential information on the composition of saffron cultivated in Northern Lebanon and highlighted the impact of geographical origin on the variability of spice composition. Variances in the main metabolite proportions were found in all samples that originated from different locations (Lebanon, Iran, Italy, and India). Our results corroborate the other studies, showing modifications to the chemical constituents of saffron extracts of different geographical origins [9] and variability between distant sites of the same territory [23]. Additionally, the results indicate a distinction between Lebanese spice from saffron of other origins, particularly the Iranian saffron, cultivated in a close area. According to the PCA model, Lebanese samples from the Qaa and Hermel regions clustered closely and had the highest scores, although these were not identical to the Iranian sample. Samples that originated from Italy and Baalbeck were matched together in a central cluster. The Indian sample showed a distinct profile, with the lowest score, indicating a possible adulteration of the spice.

Dissimilar but relatively high amounts of picrocrocin and crocins have a relevant role in the differentiation of the Lebanese spice in the tested sites. Abundant amounts of D-glucose (α and β forms), together with saturated and unsaturated fatty acids, are among the characteristic markers of Lebanese saffron. According to the specifications, the most important indicator of saffron quality is the amount of picrocrocin and crocins (ISO 3632-1 2003). Hence, the highest proportions found in the Lebanese saffron enable its general classification among the finest saffron spices.

The main differences between Lebanese saffron samples are the relative amounts of picrocrocin and the sum of different crocetin glycosides. Thus, saffron cultivated in Hermel (Leb2) seems to be the richest in these metabolites, followed by Qaa (Leb1), whereas Baalbeck saffron (Leb3) showed decreased proportions. This result can be explained by the pedoclimatic differences within these Lebanese sites. Regardless of the fact that the three regions are located at different altitudes in North Beqaa, the soil in Qaa is muddy, argillaceous and well-drained, whereas Hermel soil is stony and the soil in Baalbeck is argillaceous (personal observations). One of the best soils for saffron production has been reported to be well-drained clay-calcareous soil [24], as in the Hermel region. Additionally, hot and arid weather occurs in Qaa, compared to the humid climate in Hermel and the colder climate in Baalbeck. It has been shown that saffron cultivation sites with a higher air temperature and without excessive rain during the flowering period generated the most high-quality stigma traits [25]. The authors showed that crocins were correlated positively with air mean temperature. Hence, the fresh temperatures in the Baalbeck region could be responsible for the relatively lower proportions of the main metabolites recorded in this study, particularly for crocins. Our results corroborate with others showing the differentiation of saffron spices produced in close sites of Central Italy, which was mainly attributed to the different content of the most abundant crocins [23]. Furthermore, it is well-known that the drying procedure after harvest could influence saffron composition [15,26]. It is expected that producers refer to locally developed expertise, so drying temperature and exposure time could differ slightly between the production sites. It has been reported that saffron samples that are dried naturally show differences in aroma, and this variability was not obtained when samples were dried in the oven [27].

We previously reported the presence of polyphenols in the Lebanese saffron and their antioxidant activity against free radicals in vitro [2]. In this study, we compared phenolic amounts and scavenging properties between different saffron samples. Despite the highest amounts of picrocrocin and crocins being found in the sample of Hermel (Leb2), the saffron extract from Qaa (Leb1) was found to have the greatest values of total polyphenols, as well as strong free radical scavenging activity using ABTS and DPPH tests, similar to the Iranian sample and slightly more efficient. These observations are consistent with other studies presenting antiradical activity in the saffron extract [2,28]. To confirm the biological activity of samples, we evaluated their potential to induce a cytotoxic effect in lung cancer cells (A549). The saffron extract inhibited A549 cell growth in a dose-dependent manner (Table 2). The highest cytotoxicity occurred at 72 h, at 40 µg/mL. Our results corroborate with the previous study [29] showing that the proliferation of lung cancer cells (A549) was decreased after treatment with saffron extract, and anticancer activity was associated with the induction of apoptosis. However, the authors used high doses of saffron extract (100 µg/mL) to demonstrate cell growth inhibition and a cytotoxic effect at 72 h. In our experimental conditions, a low dose of saffron extract (10 µg/mL) was sufficient to inhibit A549 cell proliferation, causing detrimental effects and significant cell injury. Several studies have shown that the antioxidant activity of saffron could be accredited to its phenolic compounds, in addition to other active compounds like crocin, crocetin and safranal [30]. We can speculate that the anti-proliferative characteristic of saffron extract can be linked to the high amount of polyphenols and its antioxidant activity, as we noticed a correlation between polyphenols concentration in the samples, its antioxidant activity and the decrease in cell viability. Natural compounds like polyphenols are known to exhibit antioxidant activities [31,32] and have been shown to possess a wide variety of anticancer effects by inducing apoptosis and blocking cell proliferation [33,34].

Overall, the results of this investigation confirm the high specificity and great quality of Lebanese saffron based on its chemical composition and variability among the Lebanese regions, which is most probably related to the territory and the environmental factors. This study highlights the presence of large amounts of polyphenolics, combined with antioxidant activity and antiproliferative abilities in the A549 cancer cell line.

## 4. Materials and Methods

### 4.1. Saffron Samples

Saffron samples (300 mg) produced in three different areas of Lebanon were obtained directly from local producers, after being harvested between 2015 and 2017 (Table 3). The Italian saffron (three different regions) and samples from Iran and India were purchased from local producers and markets for comparison. In the case of Lebanese samples, all stigmas were collected and dried at ambient temperature, then stored in a dry and dark place until used. All samples were kept at 4 °C in the dark after delivery, until analyzed.

### 4.2. Conventional Extraction

A dried and ground sample (2 g) of stigmas from *Crocus sativus* L. was extracted with 40 mL of methanol by maceration for 24 h at ambient temperature. After filtration with Whatman paper, the plant extract was transferred into a 50 mL bottom flask, then taken with a rotary vacuum evaporator. These crude extracts were freeze-dried by lyophilization. Mass yield of saffron extract from *Crocus sativus* was 7.5% *w*/*w*. The products of conventional extraction were used for total phenolic content measurements, DPPH, ABTS, and cell viability assays.

### 4.3. Microwave-Assisted Extraction (MAE)

Dried samples were ground manually in a mortar and sifted to obtain a uniform granulometry before performing the extraction. MAE was carried out by an automatic Biotage Initiator 2.0 (Uppsala, Sweden; 2.45 GHz high-frequency microwaves, power range: 0–300 W). The internal vial temperature was controlled by an infra-red sensor probe. Manually ground saffron (10 mg) was placed in a 10 mL sealed vessel, suitable for an automatic single-mode microwave reactor, and 0.8 mL of deuterated methanol (CD_3_OD, 99.8 atom% of deuterium, Euriso-Top, France) was added to the sample to form a yellow–orange suspension. The sample was heated by microwave irradiation for 30 min at 40 °C, followed by cooling with pressurized air. An internal standard (3-(trimethylsilyl)-propionic-2,2,3,3-d_4_ acid sodium salt, TSP, 1 mM) was added, and the suspension was stirred magnetically for 1 min in the dark. The liquid phase was carefully separated from the solid precipitate and directly analyzed by NMR [9].

### 4.4. High Resolution NMR Measurements

The NMR spectra of saffron samples were recorded in CD_3_OD at 27 °C on a Bruker AVANCE 600 NMR spectrometer, operating at a proton frequency of 600.13 MHz and equipped with a Bruker multinuclear z-gradient inverse probe, producing gradients in the z-direction with a strength of 55 G cm^−1^. ^1^H spectra were referenced to the methyl signal of TSP at 0.0 ppm.

^1^H spectra of samples were acquired by co-adding 128 transients with a recycle delay of 9 s, using a 45° pulse, and 32 K datapoints. Presaturation of the residual water signal was conducted with a soft pulse of 2 s just before the acquisition. A modified zgpr Bruker pulse sequence was applied.

### 4.5. Measurement of Metabolites and Statistical Analysis

Integrals of the selected ^1^H resonances were measured with respect to the integral of TSP CH_3_ signal at 0.0 ppm, normalized to 100, and used as the internal standard. Molar concentrations of extracted metabolites in methanol were calculated according to Equation (1)

(1) $$C=C_{TSP}\frac{9}{n}\frac{I}{I_{TSP}}$$

where *C* and *C_TSP_* are molar concentrations of the selected component and TSP, correspondingly; I and I_TSP_ are integrals of the corresponding signals; n is the number of equivalent protons in the functional group of selected components. The selected resonances are listed in Table 4.

The statistical processing of NMR data was carried out using the STATISTICA package for Windows (version 5.1). Before performing the statistical analysis, the selected variables were mean-centered, and each variable was divided by its standard deviation (autoscaling). Principal component analysis (PCA) was performed on the 12 selected variables. The percentage of variance for each specific principal component was reported. PCA results are shown, reporting the scores of principal components and a plot of variable loadings.

### 4.6. Total Phenolic Content

Polyphenols have an important antioxidant activity, and their content can be determined by measuring the Folin-Ciocalteu index. Thus, the determination of phenolic compounds was based on the Folin-Ciocalteu reagent method. This reagent consists of a mixture of phosphotungstic acid and phosphomolybdic acid. The oxidation of phenols reduces this to a mixture of blue oxides of tungsten and molybdenum. The intensity of the produced color has a maximum absorption at 725 nm. This is proportional to the level of oxidized phenolic compounds and, thus, to the quantity of polyphenols present in the extracts.

First, 50 mg of the methanolic extract of each sample of saffron was dissolved in 500 mL of distilled water to obtain a stock solution of 100 µg/mL. The Folin-Ciocalteu method consists of successively introducing 0.5 mL of the diluted saffron solution, 2.5 mL of the Folin-Ciocalteu reagent, 5 mL of sodium carbonate (35% *w*/*v*) and distilled water into a 100 mL volumetric flask. This is incubated for 1 h in the dark, at an ambient temperature, and then the absorbance at 725 nm is measured by a Jenway 6405 UV-Visible spectrophotometer against a blank, where the saffron extract is replaced by water. Values were expressed in mg/L equivalents of gallic acid (GAE). The calibration line is prepared from a pure solution of gallic acid (100 mg/L) using different concentrations (10, 20, 30, 40 and 50 mg/L). The preparation of the standard solutions is similar to that of saffron solutions, but gallic acid is used instead of saffron.

### 4.7. DPPH (1,1-Diphenyl-2-Picryl-Hydrazyl Radical) Assay

DPPH solution was prepared by dissolving 32 mg of DPPH in 1 L methanol, 80%. Three milliliters of DPPH solution were added to 1 cm plastic cuvette, followed by the addition of 200 μL of the test sample (initial concentration 100 µg/mL) at increasing concentrations (150 ppm, 1500 ppm and 15,000 ppm), leading to the final concentrations of 10 ppm, 100 ppm and 1000 ppm, respectively. The mixture was shaken well and kept in the dark at room temperature for one hour. Absorbance was measured at 517 nm. Absorbance of 80% methanol was considered as a blank, while the negative control (DPPH solution) was run simultaneously. Trolox was used as a positive control. Trolox equivalent antioxidant capacity (TEAC) values were calculated using the standard regression curve.

### 4.8. ABTS^+^ Assay

ABTS^+^ was dissolved in water (7 mM) to obtain the stock solution. ABTS radical cation was produced by reacting the stock solution with 2.45 mM (final concentration) potassium persulfate solution. The solution was kept in the dark at room temperature for 12 h prior to use. Solution was diluted 50-fold with phosphate buffer (pH 8.04) and absorbance was set as 0.7 at 415 nm. Three milliliters of ABTS^+^ solution was added to 1 cm cuvette, followed by the addition of 3 μL, 15 μL and 30 μL of methanolic solution of saffron extracts (100 µg/mL) to obtain the final concentrations of 1 ppm, 5 ppm and 10 ppm, respectively. Trolox was used as a positive control, while ABTS^+^ solution served as the negative control. Absorbance was measured at 415 nm.

### 4.9. Cell Viability Assay and Statistical Analysis

A549, a human lung adenocarcinoma cell line, was maintained in Dulbecco’s modified Eagle’s medium (DMEM) supplemented with 10% fetal bovine serum (FBS), 100 units/mL penicillin and 100 µg/mL streptomycin. The cells were maintained in a humidified incubator at 37 °C and 5% CO_2_. Cell viability was measured using the 3-(4,5-dimethylthiazol-2-yl)-2,5-diphenyl-2H-tetrazolium bromide (MTT) assay. A549 cells were plated at a density of 1 × 10^3^ cells/mL in 96-well plates and allowed to attach for 24 h. Saffron extract at different concentrations (10, 20, 40 μg/mL) was added to the wells for 72 h. After treatment with saffron extracts, 10 μL of the MTT solution was added and incubated at 37 °C for 4 h. The solution was removed, followed by the addition of 100 μL dimethyl sulfoxide (DMSO) to dissolve the precipitate. Absorbance was measured at 550 nm using an automated microplate reader (Bio-Rad 550). Cell viability was expressed as a percentage of the control culture value.

Statistical analysis was carried out using ANOVA. Significant differences were determined by one-way analysis of variance test at the level of *p* < 0.05, followed by Tukey’s test using SPSS (version 16.0).

## Figures and Tables

**Table 1:** Polyphenol content and antioxidant capacity by ABTS and DPPH radical scavenging assays.

Sample Code	Geographical Origin	Total Polyphenols	DPPH	ABTS
		mg of Gallic acid/g Dry Extract	Absorbance (% Inhibition vs. ctrl)	Absorbance (% Inhibition vs. ctrl)
Leb1	Lebanon	7.2 ± 0.7 ^a^	58.5 ± 4.6 ^b^	67.5 ± 6.4 ^b^
Leb2	Lebanon	6.5 ± 0.9 ^a^	42.3 ± 3.9 ^a^	46.4 ± 5.4 ^a^
Leb3	Lebanon	6.2 ± 0.6 ^a^	47.8 ± 5.8 ^ab^	41.2 ± 3.9 ^a^
Iran	Iran	7.1 ± 0.7 ^a^	59.9 ± 5.8 ^b^	65.5 ± 5.8 ^b^
India	India	5.8 ± 0.7 ^a^	47.8 ± 3.5 ^ab^	47.8 ± 3.4 ^a^

40 μg/mL of each saffron sample was used. The results are expressed as mean ± SD (*n* = 3) with a 95% confidence interval. Means followed by different superscript letters (^a^ vs. ^b^) are significantly different at *p* < 0.05, whereas means followed by ^ab^ are not different from ^a^ or ^b^ ones.

**Table 2:** Effect of saffron samples on A549 cell viability.

Origin of Samples	Concentration (μg/mL)	% Cell Viability (72 h)
Lebanon	10	75 ± 4.2 ^c^
	20	42 ± 3.9 ^b^
	40	25 ± 2.8 ^a^
Iran	10	72 ± 3.7 ^c^
	20	45 ± 3.0 ^b^
	40	22 ± 3.6 ^a^

Percentage of cell viability was determined using MTT assay. Cells were incubated with different concentrations of saffron for 72 h. Cell viability was expressed as a percentage of the control culture value (100%). The results are shown as mean ± SD (*n* = 3). Means followed by different superscript letters (^a^ vs. ^b^ vs. ^c^) are significantly different at *p* < 0.05.

**Table 3:** Saffron samples analyzed by NMR.

Sample Code	Geographical Origin
Leb1	Lebanon, Qaa
Leb2	Lebanon, Hermel
Leb3	Lebanon, Baalbeck
Iran	Iran
India	India
Ita (AB)	Italy, Abruzzo
Ita (LA)	Italy, Lazio
Ita (SA)	Italy, Sardinia

**Table 4:** Selected ^1^H NMR signals in the NMR spectra of saffron extracts.

Metabolite	Annotation	Group	*n*, Number of Equivalent Protons	^1^H Chemical Shift, ppm/Multiplicity ^a^
Fatty acids (FA)	2	CH_3_	3	0.90 /m
Picrocrocin (PCROC)	3	CH_2_-5	1	1.85 / ddd: [12.6; 3.3; 2.2]
Acetic acid (AcOH)	4	CH_3_	3	1.94 /s
Linoleic fatty acid (C18:2)	5	CH_2_-11	2	2.78/ t: [6.8]
Linolenic fatty acid (C18:3)	6	CH_2_-11, 14	4	2.81/ t: [6.8]
β-Glucose (βGLC)	7	CH-2	1	3.12 /dd: [9.2; 7.8]
α-Glucose (αGLC)	9	CH-1	1	5.10 /d: [3.7]
Unsaturated fatty acids (UFA)	10	CH=CH	2	5.34 / m
β-D-gentiobiosylcrocins (GB-Croc)	8	CH_2_-6	1	4.17 /dd: [11.5; 2]
β-D-glucosylcrocins (βGLC-Croc)	11	CH-1	1	5.56 / d: [7.7]
All-*trans*-crocetin (DBtCroc), aglycone moiety	12	CH=CH-10,10′	2	7.44 / dm: [11.3]
13-*cis*-crocetin (DBcCroc)	13	CH-10	1	7.51 /d: [11.8]

^a^ d—doublet, t—triplet, m—multiplet, s—singlet; J constant (in Hz) are also reported in square parenthesis.

## Data Availability

The data presented in this study are available on request from the corresponding author.

## Sample Availability

Samples of saffron are available from the authors.
